# Expanding a Health Technology Solution to Address Therapist Challenges in Implementing Homework With Adult Clients: Mixed Methods Study

**DOI:** 10.2196/56567

**Published:** 2024-12-12

**Authors:** Brian E Bunnell, Kaitlyn R Schuler, Julia Ivanova, Lea Flynn, Janelle F Barrera, Jasmine Niazi, Dylan Turner, Brandon M Welch

**Affiliations:** 1 Department of Psychiatry and Behavioral Neurosciences Morsani College of Medicine University of South Florida Tampa, FL United States; 2 Department of Psychology University of North Carolina Wilmington Wilmington, NC United States; 3 Doxy.me Research Doxy.me Inc Rochester, NY United States; 4 Department of Public Health Sciences Medical University of South Carolina Charleston, SC United States

**Keywords:** mental health, mental illness, mental disease, mental disorder, homework, homework challenge, therapy, therapist, barriers, adult client, adult, technology-based solution, health technology, digital health, digital technology, digital intervention, mobile phone

## Abstract

**Background:**

Homework is implemented with variable effectiveness in real-world therapy settings, indicating a need for innovative solutions to homework challenges. We developed Adhere.ly, a user-friendly, Health Insurance Portability and Accountability Act–compliant web-based platform to help therapists implement homework with youth clients and their caregivers. The initial version had limited functionality, was designed for youth clients and their caregivers, and required expanding available features and exercises to suit adult clients.

**Objective:**

The purpose of this study was to better understand barriers and potential solutions to homework implementation experienced by therapists seeing adult clients and obtain their input on new features and exercises that would enable Adhere.ly to better meet their needs when working with this population.

**Methods:**

This study used an exploratory, sequential mixed methods design that included 13 semistructured focus groups with mental health therapists and clinic leaders and a survey administered to 100 therapists. Analyses were performed using the NVivo qualitative analysis software and SPSS.

**Results:**

The findings revealed common barriers, such as clients and therapists being busy, forgetting to complete homework, managing multiple platforms and homework materials, and clients lacking motivation. Adhere.ly was perceived as a potential solution, particularly its user-friendly interface and SMS text-message based reminders. Therapists suggested integrating Adhere.ly with telemedicine and electronic health record platforms and adding more exercises to support manualized therapy protocols and therapy guides.

**Conclusions:**

This study highlights the importance of technology-based solutions in addressing barriers to homework implementation in mental health treatment with adult clients. Adhere.ly shows promise in addressing these challenges and has the potential to improve therapy efficiency and homework completion rates. The input from therapists informed the development of Adhere.ly, guiding the expansion of features and exercises to better meet the needs of therapists working with adult clients.

## Introduction

### Background

Mental health disorders affect approximately 20% of individuals in the United States and are associated with costly physical, social, and occupational problems [1-3]. Most evidence-based mental health treatments (eg, cognitive behavioral therapy [CBT]) involve introducing and practicing therapeutic mental health exercises during therapy sessions, assigning patients to practice those therapeutic exercises between therapy sessions, and reviewing that practice during the next therapy session [4,5]. This between-session practice, or homework, facilitates treatment processes, strengthens learning, and enables the skills learned in therapy to generalize to the client’s everyday environment, leading to improved maintenance of treatment gains [6-8].

Despite its many benefits, homework is implemented with variable effectiveness in real-world clinical settings. Only 68% of general mental health care providers and approximately 55% of family care providers report using homework “often” to “almost always” [9,10]. Therapists report using homework in an average of 57% of sessions, and only 25% of therapists report using expert-recommended systematic procedures for implementing homework (ie, specifying frequency, duration, and location and writing down homework assignments for patients) [11]. A national survey revealed that 93% of mental health therapists estimate rates of client adherence to homework to be low to moderate [10], and studies generally report low to moderate rates of client homework adherence [12,13].

Previous research has outlined numerous barriers to the successful implementation of homework during mental health treatment [6,7,9,14-21]. In our prior work, we found that youth therapists often have difficulty consistently and effectively designing, introducing, practicing, assigning, and assessing homework exercises, especially given the time constraints and workload of everyday clinical practice. We also found that youth and caregiver clients often have difficulty remembering to complete assignments and understanding the rationale and practical implementation (ie, when, where, and how) of homework exercises [14]. As part of this prior work, we also explored potential health technology solutions to these barriers. Therapists and youth and caregiver clients suggested digitized therapeutic exercises with instructions for clients, automated SMS text message–based reminders to complete those therapeutic exercises for homework, behavior and symptom tracking, reports, and activity summaries. Therapists and clients also expressed beliefs that a health technology solution with these features would likely increase therapist use of and client adherence to homework and have positive effects on the therapeutic relationship, treatment efficiency, and treatment effectiveness [14].

Informed by these results and using an iterative, user-centered design approach, we developed Adhere.ly, a user-friendly, Health Insurance Portability and Accountability Act–compliant web-based platform to help youth therapists implement homework with youth clients and their caregivers [22]. Upon initial development, the major components of Adhere.ly were designed to help youth therapists (1) *practice* digitized therapeutic mental health exercises (eg, relaxation, affect and emotion, cognitive, exposure, and parent-child activities) with their youth and caregiver clients in the session, (2) *remind* clients to practice homework between therapy sessions via automated SMS text message or email messages with links to those exercises to be opened and completed in the clients’ smartphone or computer browser, and (3) *review* clients’ homework completion and relevant data for certain exercises (eg, self-monitoring ratings and anxiety ratings during exposure exercises). Adhere.ly is accessed via computer, tablet, and smartphone browser and does not require any downloads by clients or therapists. Clients do not create accounts or need to log in to complete exercises assigned by therapists.

### Objectives

The initial version of Adhere.ly included limited functionality and exercises designed solely for youth clients and their caregivers. Although therapeutic approaches with children and adults share many theoretical foundations, such as CBT, there are numerous developmental considerations that must be taken into account when providing therapy to children and adolescents [23,24]. Some of these include the focus and goals of the therapy (eg, individual vs systemic), the involvement of others (eg, spouses vs caregivers), and the way in which therapeutic concepts and techniques are communicated (eg, didactic vs gamified) [25]. As we sought to expand Adhere.ly to support therapists in treating adult mental health clients, we felt that it would be critical to obtain the input of these key stakeholders. The first step toward accomplishing this goal was to better understand barriers and potential solutions to homework implementation experienced by therapists seeing adult clients. Next, we sought to obtain their input on new features and exercises that would enable Adhere.ly to better meet their needs when working with this population.

## Methods

### Overall Design

We used an exploratory, sequential (qualitative then quantitative) mixed methods design to understand (qualitative) and prioritize (quantitative) (1) the challenges and goals of stakeholders relative to the use of health technology solutions such as Adhere.ly and (2) new features and exercises to support therapists delivering evidence-based therapy to adult clients. The qualitative component of this design included remote (ie, videoconference or telephone), ≤90-minute screen- and audio-recorded semistructured focus groups with mental health therapists and clinic leaders. The quantitative component included a survey among mental health therapists.

### Ethical Considerations

This study was approved by the University of South Florida Institutional Review Board (STUDY002555). For the focus groups, we scheduled interested individuals and obtained individual verbal consent and demographic information before starting the group interview. For the survey, after following the link and completing a screener, participants clicked a button to indicate their electronic informed consent to participate. All focus group and survey data were deidentified before analysis. Focus group participants received a US $50 gift card for their time, and we compensated participants with a US $50 gift card for their time upon completion of the survey.

### Focus Groups

#### Participants and Recruitment

We conducted a total of 13 focus groups with mental health therapists (n=18) and clinic leaders (n=16) between September 1, 2021, and October 25, 2021. A range of 2 to 6 mental health therapists and exactly 2 clinic leaders were included in each of the 8 clinic leader focus groups, and therapists and clinic leaders were interviewed separately. We did not meet our initial recruitment goal of 24 mental health therapists and 24 clinic leaders due to recruitment challenges during the COVID-19 pandemic.

All focus group participants were English-speaking adults (aged ≥18 years). We included therapists who had obtained at least a master’s degree in social work, counseling, clinical psychology, or a related field and carried active adult mental health treatment caseloads. We included clinic leaders who served in a supervisory, managerial, or ownership role for an organization focused on providing mental health treatment services. Research staff emailed mental health therapists and clinic leaders registered with Doxy.me, a commercial telemedicine platform, with information about the study and an invitation to participate.

#### Data Collection and Instruments

In total, 2 master’s-level and 1 bachelor’s-level research staff members trained in social and behavioral sciences research facilitated the focus groups, and all 3 are authors on this manuscript. The focus group facilitators were university employees and not employed by Adhere.ly, LLC. Facilitators were trained with a detailed semistructured interview guide and protocol, including a series of role-play exercises and strategies to minimize potential bias. The focus group semistructured interview guide was developed following the Consolidated Framework for Implementation Research (CFIR) codebook [26] and the Integrated Technology Implementation Model (ITIM) [27]. All the interview questions are included in Multimedia Appendix 1. All focus groups were recorded and transcribed using the Dovetail transcription software (Dovetail Research Pty Ltd). Transcriptions were reviewed and edited to ensure accuracy while listening to the recordings.

The semistructured focus groups began with questions about challenges that therapists experience while trying to engage adult clients in homework during therapy and strategies that therapists have used to try to overcome those challenges. Next, participants viewed a 5-minute video tutorial on Adhere.ly and its current exercises and features. We then asked participants to share their perspectives on how Adhere.ly might address challenges to engaging clients in homework and for their input on changes that might be made to Adhere.ly to meet the needs of therapists treating adult clients, including the addition of new features and therapeutic exercises.

#### Data Analysis

The qualitative analysis was completed by a team of 4 research staff members, 3 of whom are authors on this manuscript. All research staff members had training in sociobehavioral sciences, including 2 with doctoral degrees in the social sciences (ie, clinical psychology and health and human performance), 1 with a master’s degree in psychology, and another with a bachelor’s degree in psychology. In total, 2 members of the analysis team also facilitated the focus groups. The researchers coded the focus group recordings using NVivo (version 11; QSR International), a qualitative analytical platform. Procedures followed the COREQ (Consolidated Criteria for Reporting Qualitative Research) [26]. The team read through the transcripts line by line to identify sensitizing categories reflective of the data. Researchers used a hybrid deductive-inductive process using thematic analysis [27,28]. The researchers coded emerging categories based on prior work that informed the focus group questions [14,29-31]. The unit of analysis was meaningful phrases [27,28].

Specifically, the team completed an initial round of deductive coding using categories identified through (1) prior work evaluating barriers to implementing homework through telemedicine [14,25] and (2) the CFIR and ITIM frameworks [26,27]. Categories related to homework challenges and requested exercises and features were created using evidence from previous studies on the platform [14,25], including but not limited to motivational and internet access–related issues and care provider attitudes toward homework. Categories following the CFIR and ITIM frameworks included but were not limited to the adaptability, compatibility, complexity, efficiencies, and relative advantages of Adhere.ly.

Results of this initial round were used to develop survey questions that asked respondents to priority rank the therapist- and client-related barriers to homework and the requested features and exercises identified in the first round of coding.

During the second round of coding, researchers refined the codebook over 4 iterations of inductive coding to identify categories, and disagreements were resolved through consensus with the research team. The research team coded a third of the focus groups collaboratively until the Cohen κ was at least 0.61, indicating substantial agreement, and the remaining focus groups were coded independently. Focused coding was used to refine the coding and ensure that the data were coded completely with minimal redundancy [32]. A full list of categories and the number of associated references are provided in Multimedia Appendix 1.

### Survey

#### Participants and Recruitment

We administered the 15-minute, web-based survey among 100 English-speaking adult (aged ≥18 years) mental health therapists who had obtained at least a master’s degree. Similar to the recruitment of focus group participants, research staff emailed mental health therapists registered with Doxy.me, including those who had participated in the focus groups, with information about the study, an invitation to participate, and a link to the survey screener. Survey responses were collected between October 21, 2021, and December 21, 2021.

#### Data Collection and Instruments

We developed a 59-question survey using insights obtained from the focus groups and the literature on homework and process-based CBT [33]. The survey began with screening questions about age, specialty (eg, mental health counselor vs social worker vs psychologist), and education (ie, master’s degree vs doctoral degree vs neither), followed by questions about personal (ie, sex, race, and ethnicity) and professional (eg, ages and disorders treated, practice type, primary reimbursement, and treatment paradigms followed) demographics. The survey then asked therapists questions related to their use of homework with their adult clients. These included questions about how often they asked clients to complete homework exercises using a 4-point Likert scale (ie, 1=*never*; 4=*most of the time*), how important they thought homework was to improving their clients’ outcomes on a 5-point scale (ie, 1=*not at all*; 5=*extremely*), and how important it was for them to know the results of their clients’ homework assignments (eg, whether they completed them and how it went) on the same 5-point scale (ie, 1=*not at all*; 5=*extremely*). The survey also included questions about the extent to which certain factors were a barrier to them using homework with their clients (eg, not knowing what or how to assign homework, forgetting, and being too busy) and their clients completing homework assignments (eg, not knowing why or how to do the homework assignment or what to do, forgetting, being too busy, and avoiding homework due to distress) using a 4-point scale (ie, 1=*not a barrier*; 4=*significant barrier*). Therapists then viewed a 5-minute video tutorial on Adhere.ly and its current exercises and features. Afterward, the survey asked them to rank a list of exercises (eg, relaxation, interpersonal, self-monitoring, and behavioral activation) and a separate list of features (eg, assessments, integration with telemedicine platforms, integration with electronic health record [EHR] software, and therapy guides) in the order in which they should be prioritized when added to Adhere.ly to meet therapists’ needs when seeing adult clients. All survey questions are included in Multimedia Appendix 2.

#### Data Analysis

We cleaned the survey data and used SPSS (version 28; IBM Corp) to calculate descriptive statistics. We averaged the rank order of the features and exercises and used mean rankings to determine relative priority.

## Results

### Focus Groups

#### Participants

The demographic characteristics of the focus group participants are shown in Table 1. On average, participants were aged 47.97 (SD 13.21) years and were largely female (25/34, 74%), White individuals (23/34, 68%), and non-Hispanic or Latino individuals (31/34, 91%). As shown in Table 1, demographic characteristics were similar for the therapist and clinic leader groups.

**Table 1:** Focus group participant demographics (N=34).

Demographics		Overall sample	Therapists (n=18)	Clinic leaders (n=16)
Age (y), mean (SD)		47.97 (13.21)	49.7 (15.80)	46.1 (9.60)
Sex (female), n (%)		25 (74)	12 (67)	13 (81)
**Race, n (%)**				
	Asian	1 (3)	0 (0)	1 (6)
	Black	8 (24)	5 (28)	3 (19)
	Multiracial	2 (6)	2 (11)	0 (0)
	White	23 (68)	11 (61)	12 (75)
Ethnicity (non-Hispanic or Latino), n (%)		31 (91)	16 (89)	15 (94)

The therapist and clinic leader focus groups were analyzed together, and these data were reported in a unified coding scheme comprising categories of responses. The number of therapists and clinic leaders that made statements relevant to each category is reported in Multimedia Appendix 1. In addition, illustrative quotes are provided for each category. It should be noted that only therapists and clinic leaders were participants, so references made to client-related barriers are therapist and clinic leader perceptions of those client barriers.

#### Challenges to Engaging Clients in Homework

Participants identified 7 major categories related to challenges in engaging clients in homework (44 total references). Overall, most of the focus group participants reported challenges related to therapists being too busy to complete, assign, or review homework (9/44, 20% of references) or perceiving that their clients were too busy to complete homework (8/44, 18% of references). However, the number of participants who made statements within both categories differed substantially between the clinic leader and therapist focus groups.

One participant commented the following:

We have not had much luck with getting anything returned to us, whether it’s homework or releases of information...

Another participant stated the following:

...as an individual provider or even in a clinic, you don’t have the time to be constantly checking in every day with all the clients to get them to practice what you’re telling them to practice.

Most of the references associated with the category of therapists being too busy to assign homework and perceptions that clients were too busy to complete homework were from the clinic leader focus groups (7/9, 78% of references) as opposed to the therapist focus groups (2/9, 22% of references). Slightly more clinic leaders (5/8, 62% of references) than therapists (3/8, 38% of references) endorsed perceiving that clients forget to complete homework. While these were the 2 most common categories of responses in the clinic leader focus groups, neither was the most frequent in the therapist focus groups.

In addition, many focus group participants reported struggling to find the right platform or medium for homework (7/44, 16% of references). The number of relevant responses did not differ substantially between the therapist (3/7, 43% of references) and clinic leader (4/7, 51% of references) focus groups. Participants reported difficulty sending homework materials over telemedicine platforms as well as having to manage multiple web-based platforms and applications to meet their clients’ needs.

One participant commented the following:

When I know that I’m going to be assigning homework assignments, I’ll have the worksheet or paper in front of me so that I can give it to them. We use Doxy.me for our tele-health and it’s a little hard, I can sometimes screen-share, but sometimes our tele-health is over the phone and I’m like, okay, just try and Google this on your own. Good luck. It makes it a little challenging.

The next 2 most frequently reported challenges included perceptions that clients lack motivation or discipline to complete homework consistently (7/44, 16% of references) and perceptions that clients receive insufficient instruction or rationale for completing homework (6/44, 14% of references). A comparable number of therapists and clinic leaders made statements that fell under these 2 categories.

One participant commented the following:

I think when it comes to giving a client an assignment, it’s always a challenge as to whether they’re going to follow through with it and utilize it. And also, I mean, at least they’ll try it once and whether they’re going to try it again, will depend on if it worked or not, or if it was so empowering to them that they’re willing to keep using it.

Few participants mentioned challenges related to perceptions of clients avoiding homework due to anticipated distress (4/44, 9% of references) and clients experiencing external (ie, internet or phone access), educational, or cognitive functioning barriers to completing homework (3/44, 7% of references).

For example, one participant stated the following:

So, like you’re in therapy and you’re in the moment...and then in between sessions, you just sort of like, don’t want to go there. So, you avoid.

Another participant stated the following:

I would also add that we have some resource barriers here sometimes. So, I’m in a fairly rural part of the country. And as a result of that, the ability to obtain a consistent internet or phone signal sometimes is really challenging. So, there’s some technological barriers in that regard.

Related to educational or cognitive barriers to completing homework, another participant stated the following:

And then I also think the education and cognitive abilities of the person sometimes plays a role in making sure that the materials we’re providing are appropriate for the person. So, if I try giving them really complicated interventions and I send them home with a sheet and their reading isn’t strong enough to be able to comprehend what the sheet says that can also be a barrier in some situations.

While the category related to client avoidance was evident in both the therapist (1/4, 25% of references) and clinic leader (3/4, 75% of references) focus groups, the category related to socioeconomic status, rural versus urban location, educational level, or cognitive ability challenges was evident only in the clinic leader focus groups (3/44, 7% of references).

Overall, most clinic leaders emphasized therapists’ and clients’ busy schedules and perceptions that clients forget to complete their homework. Most therapists emphasized perceptions that clients lack motivation or discipline to complete homework consistently; however, the number of participants whose responses fell within these categories was more evenly distributed across categories in the therapist focus groups than in the clinic leader focus groups.

#### Current Strategies for Overcoming Challenges

Participants’ responses fell into 5 categories of strategies or responses that therapists used to overcome challenges in engaging clients in between-session exercises (57 total references). Most participants referenced using motivational and reinforcement strategies to encourage homework completion during the sessions (23/57, 40% of references); however, nearly all the participants who mentioned this strategy were clinic leaders (19/23, 83% of references) as opposed to therapists (4/23, 17% of references):

We have to really just plan out how important the assignments are to their treatment, and just for them to know that if they want to get better and achieve the goals that we’ve set in therapy, then the homework assignments are a very important part of that, and they need to take it seriously.

Many participants also mentioned using multiple applications and web platforms to send and track homework (15/57, 26% of references). Similar to the previous category, most participants who mentioned this strategy were clinic leaders (13/15, 87% of references) rather than therapists (2/15, 13% of references):

...some of us have gotten creative. We realized that the paper homework doesn’t work...or having the client record their thoughts on their phone or on a paper does not work. So, we get written permission from the client to share Google Drive, which is probably not the most HIPAA compliant activity to be doing. We have to go through the whole hassle of getting the client to sign that they’re okay with this and that they’re sharing that Google document. Knowing that the therapist is able to review it and see it in real time tends to have a little bit of an effect on compliance.

The third most frequently referenced strategy was adapting the treatment plan to “meet clients where they are at,” which largely entailed changing homework assignments or completing them in the session rather than continuing to give the same homework assignments that the clients had not previously completed (14/57, 25% of references). This category was equally referenced by clinic leaders (7/14, 50% of references) and therapists (7/14, 50% of references):

I don’t penalize and you know, I don’t shame or anything like that when people don’t do their homework. However, like a good teacher, if you don’t do your homework, you’re doing it in class. And so, we just break out the pen and paper, I get silent, turn the lights down, if I need to, or the lights up, whatever is going to be conducive. But sometimes people, this is the one time they get a chance to really just be still and really think, cause they’re on autopilot so much.

The last 2 strategies were referenced by only a few focus group participants and included having clients set their own reminders either electronically or in written form (3/57, 5% of references), which only emerged in the clinic leader focus groups, and providing accountability via email, SMS text message, or phone (2/57, 4% of references), which was mentioned by only 1 participant in the clinic leader and therapist focus groups each. For example, one participant commented the following:

...we’ve definitely had clients who forget things that we’ve had them put it in their calendar in their phone.

#### How Adhere.ly Might Address Challenges to Engaging Clients in Homework

Participants’ responses to how Adhere.ly might address challenges to engaging clients in homework were divided into 11 categories (143 total references). Overall, the most common responses were that the utility of Adhere.ly would vary by client demographics and clinical needs (28/143, 19.6% of references), which was emphasized almost equally by therapists (18/28, 64% of references) and clinic leaders (10/28, 36% of references), and that Adhere.ly is easy to use and would appeal to a wide range of therapists and clients (27/143, 18.9% of references), which was emphasized by more therapists (12/27, 44% of references) than clinic leaders (15/27, 56% of references).

Regarding the utility of Adhere.ly varying by client demographics and clinical needs, participants mentioned demographics such as age, which they thought might influence attitudes toward technology, and smartphone use. Many participants also mentioned generally taking the approach of seeing how specific clients responded to new interventions before they drew conclusions about effectiveness and being unable to comment on Adhere.ly until they had piloted it with their clients. A smaller subset of these participants mentioned that Adhere.ly may not be helpful for specific clinical populations, including those in crisis or with serious substance abuse problems:

I know a lot of my older people...my older ladies, high anxiety, depression, they’re going to have difficulty understanding it. I mean, it’s something that I think could be very useful to a certain group of folks.

I think this also speaks to a particular demographic. I see a fair percentage of Medicare patients, some of whom don’t have smartphones, and some who are very smart about using their smartphone. So, you know, defaulting to that, it’s enough of a challenge to get people, some of them, to do telemedicine. So, we’d have to look at whether this is a demographic that is comfortable doing things on the phone.

Regarding ease of use, participants mentioned that Adhere.ly seemed more accessible and efficient than other applications and platforms, which was mentioned by nearly the same number of therapists (12/143, 8.4% of references) and clinic leaders (15/143, 10.5% of references):

I definitely agree that even compared to some other apps...the user interface seems a lot simpler. I kind of poked around before and created an account and it actually moves pretty quickly and doesn’t lag and it’s just easier to navigate.

Many participants reported perceptions that Adhere.ly would increase the overall efficiency of therapy (23/143, 16.1% of references), which was mentioned by most participants across the therapist (11/23, 48% of references) and clinic leader (12/23, 52% of references) focus groups. Several specifically mentioned that Adhere.ly’s reminder feature would improve homework completion rates (16/143, 11.2% of references), although this was primarily emphasized by clinic leaders (15/16, 94% of references) rather than therapists (1/16, 6% of references). One participant commented the following:

I think it reminds me of Fitbit. It’s kind of the same concept of getting people to get up and move or exercise and, it’s just, again, reinforcing a different habit.

Another participant stated the following:

...being able to know that there’s some real practical, useful tools and automated support reinforces the fact that folks are getting what they need in between sessions. Cause it’s absolutely difficult to have to start over again week by week because you feel like you lost what happened between the sessions.

Many participants also stated that the utility of Adhere.ly would vary by therapist demographics and theoretical orientation (12/143, 8.4% of references), although more therapists (18/143, 12.6% of references) emphasized this than clinic leaders (10/143, 7% of references). Some of these comments referenced the association between age and attitudes toward technology or attitudes toward technology alone, as illustrated by the following quotes:

I have some [therapists] that are really tech savvy. And so, this is going to seem like a breeze, and it would be pretty easy for them to incorporate it. And I have others that wish that we didn’t have electronic health records and wish we could still do pencil and paper for everything. And so, I would imagine this would probably align with comfort level in part, but as long as it was fairly user-friendly, I would imagine probably most of my clinicians would at least try it out.

I have staff under me who are in their fifties. I have staff in their thirties. So, I think it just varies. I can’t speak to everybody’s level of comfortability. That’s the best answer I can give you.

Therapists also mentioned that Adhere.ly might be especially useful when implementing evidence-based CBTs over telehealth but might be less useful if using other interventions or doing therapy in person:

It’s not as much a need for in-person services as for integrated CBT interventions on doxy.me. That feels like a need. Like, to me, that’s the missing piece. I think I mentioned this in our first meeting, even as a clinical supervisor, if I have a very beginning therapist and I’m trying to teach her how to teach the cognitive triangle or how to teach a thought record, and I’m doing it over zoom, which I did for so long.

Some participants also mentioned that Adhere.ly would easily fit into therapists’ in-session workflows (11/143, 7.7% of references), which was mentioned by slightly more clinic leaders (7/11, 64% of references) than therapists (4/11, 36% of references). Participants explained that therapists could easily set up Adhere.ly exercises with their clients at the end of the session, which would minimize the time they would need to spend doing homework outside the session:

Many times, we have patients back-to-back and so the ability to do it in between appointments isn’t always there. I think if the provider saw the value in this, I would imagine they would incorporate it into the session on the back end as kind of a wrap up between that and the following session. I think if it was like an add-on that had to be done in between sessions, that could create some challenges only because of the learning curve and time commitments.

Other less commonly referenced categories included Adhere.ly’s ability to capture treatment data (9/143, 6.3% of references) and the use of text-based communication (6/143, 4.2% of references) coming from their therapist as opposed to an external platform (5/143, 3.5% of references) to increase client engagement and homework completion. For example, one participant commented the following:

I like the tracking. I like the little charts that show you how they’re doing.

Another participant said the following:

I like that it goes straight to their email or their texts without a log-on...if there’s no login, I find that clients are much more likely to click on a link and then follow through.

Primarily clinic leaders emphasized the benefits of capturing treatment data (8/9, 89% of references) and the use of text-based communication (5/6, 83% of references), whereas more therapists emphasized the benefits of clients receiving direct communication from their therapist through Adhere.ly (4/5, 80% of references).

#### Suggested Features to Add to Adhere.ly

When asked which features should be added to Adhere.ly (63 references in total), a total of 7 categories emerged, although most of the references included in this account were from the clinic leader focus groups (52/63, 83% of references). The most common response was to add a demonstration video or other web-based walk-through for clients and therapists (14/63, 22% of references), and 86% (12/14) of these references were from the clinic leader focus groups. One participant stated the following:

...make an electronic training that actually sends the links to the clinicians so that they see what it’s like, and they’re sort of exposed to it from the client side.

This was followed by integration of Adhere.ly with EHR platforms and Doxy.me (12/63, 19% of references), which was only mentioned by clinic leaders. One participant stated the following:

I think the toughest part would be if people have to log into a separate platform...the more integrated it is into something you’re already using or the easier it is to get in and out of, the more helpful it would be.

Other suggestions included enhancement of audiovisual features (ie, gamification and more color and visuals; 11/63, 17% of references), which was primarily mentioned by clinic leaders (8/11, 73% of references) rather than therapists (3/11, 27% of references):

There’s got to be some type of draw that keeps somebody continuing to engage with electronic interventions...like gamification. For example, can they achieve ribbons or trophies or can go toward something that shows them that they’re making progress? I know at least with some of the programs that I utilize now, that’s what I really like about it.

Several participants also suggested expanding the reminder feature (ie, recurring notifications, customizations, and parent reminders; 10/63, 16% of references), which was equally mentioned by therapists (5/10, 50% of references) and clinic leaders (5/10, 50% of references). For example, a participant suggested adding “a way to set the reminder up for an open-ended period of time until it’s changed, rather than every week having to get them there and change the reminder.” Other suggestions mentioned by several participants included adding more options for registration (ie, self-registration and customized communication preferences, including more client identifiers and a feature for group, couple, and family registration; 8/63, 13% of references) and adding an e-consent form (5/63, 8% of references). One participant emphasized the following:

Share-ability! Where can I share the information? Is it with the client? Is it with their parent? Is it with a family member? Clinician share-ability I think goes a long way to making something useful.

Both of these features were only mentioned by clinic leaders.

There were several features that did not fit neatly into the other categories, all of which were referenced by only 1 participant, such as options for clients to provide feedback on exercises, a feature allowing PDF export of client results, and ensuring sixth- and seventh-grade reading level across the content. These features were also only requested by clinic leaders.

#### Suggested Therapeutic Exercises to Add to Adhere.ly

When asked which therapeutic exercises should be added to Adhere.ly, participants responded within 7 major categories (81 references in total), most of which were identified by therapists (47/81, 58% of references) These categories included the following: homework from evidence-based therapy protocols (40/81, 49% of references), homework for specific skills or strategies (17/81, 21% of references), homework for specific client demographics (6/81, 7% of references), homework for specific mental health conditions (5/81, 6% of references), personalized or custom exercises (5/81, 6% of references), and brief or single-item assessments (3/81, 4% of references).

Regarding homework for evidence-based therapy protocols, most therapists and clinic leaders identified the following interventions as useful additions: mindfulness-based approaches (11/40, 28% of references), trauma-focused therapies (ie, eye movement desensitization and reprocessing, cognitive processing therapy, and prolonged exposure; 8/40, 20% of references), dialectical behavior therapy (5/40, 12% of references), behavioral activation (4/40, 10% of references), safety planning (3/40, 8% of references), CBT (3/40, 8% of references), and acceptance and commitment therapy (2/40, 5% of references). One participant explained why they emphasized trauma-focused interventions:

Much of my focus is trauma. And so many of the approaches I use include things like EMDR and somatic experiencing. So...if I was going to assign anything to anyone, it would be related to developing self-regulation skills and somatic awareness skills to help them be able to cope with trauma symptoms. So that would be one way that I can see providing homework to people is to do something like that.

While mindfulness and stress reduction interventions were mentioned by an equal number of therapists (6/11, 55% of references) and clinic leaders (5/11, 45% of references), most of the remaining interventions were mentioned by more clinic leaders than therapists, although the overall number of suggested interventions was greater for therapists than clinic leaders.

With respect to homework for specific skills and strategies, therapists most frequently requested journaling (7/17, 41% of references), creative activities (3/17, 18% of references), recovery plans (2/17, 12% of references), and strength-based activities and positive psychology (2/17, 12% of references). These distributions can be viewed in greater detail in Multimedia Appendix 1.

One participant suggested the following:

I think those basic needs things like, are you sleeping? Are you eating? Have you had water today? For that kind of stuff, they need reminders because when they’re really depressed, as we know, it’s hard to remember those basic things.

Regarding homework for specific client demographics (5/81, 6% of references), some therapists referenced emotion regulation games that are engaging for children (3/5, 60% of references) despite the prompt to suggest interventions for adult users, as well as couples exercises (2/5, 40% of references). For example, one participant suggested the following:

...for clinicians working with couples...there are lots of lots of things like scanning for opportunities to express appreciation and respect...prompt them to initiate a conversation to show appreciation.

No clinic leaders suggested interventions for specific demographics or mental health conditions. The remaining categories, including homework for specific mental health conditions and personalized or custom exercises, did not have a pattern of minor categories and were only mentioned by a few therapists.

### Survey

#### Participants

The demographic characteristics of the survey respondents are shown in Table 2. On average, therapists were aged 45.6 (SD 14.23) years and were largely female (69/100, 69%), White individuals (79/100, 79%), and non-Hispanic or Latino individuals (85/100, 85%). Therapists were mostly mental health counselors (39/100, 39%), psychologists (28/100, 28%), and social workers (22/100, 22%) who worked in individual practice (55/100, 55%) and small clinic (33/100, 33%) settings and were primarily reimbursed via private (53/100, 53%) or public (32/100, 32%) insurance. Most therapists primarily saw adults (93/100, 93%) and adolescents (51/100, 51%) with anxiety (99/100, 99%), trauma- and stressor-related (90/100, 90%), and mood (85/100, 85%) disorders following the cognitive behavioral paradigm (69/100, 69%).

**Table 2:** Demographic and professional characteristics of the survey respondents (N=100).

Demographics			Values
Age (y), mean (SD)			45.64 (14.23)
Sex (female), n (%)			69 (69)
**Race, n (%)**			
	American Indian or Alaska Native	1 (1)	
	Asian	3 (3)	
	Black	13 (13)	
	Multiracial	11 (11)	
	White	79 (79)	
Ethnicity (non-Hispanic or Latino), n (%)			85 (85)
**Highest degree, n (%)**			
	Master’s	67 (67)	
	Doctoral	33 (33)	
**Specialty, n (%)**			
	Mental health counselor (eg, LMHC^a^ or LPC^b^)	39 (39)	
	Psychologist (eg, PhD or PsyD)	28 (28)	
	Social worker (eg, LCSW^c^)	22 (22)	
	Marriage and family therapist (eg, LMFT^d^)	9 (9)	
	Other mental health therapist	1 (1)	
**Type of clinic or organization, n (%)**			
	Individual practice	55 (55)	
	Network of health care providers or small clinic	33 (33)	
	Hospital or large clinic	10 (10)	
	Educational setting	2 (2)	
**Primary method of reimbursement, n (%)**			
	Private insurance	53 (53)	
	Public insurance (Medicare or Medicaid)	32 (32)	
	Out of pocket by the client	15 (15)	
**Age groups treated, n (%)**			
	Adults (aged 18-64 y)	93 (93)	
	Adolescents (aged 11-17 y)	51 (51)	
	Older adults (aged ≥65 y)	32 (32)	
	Children (aged 0-10 y)	26 (26)	
**Mental health disorders treated, n (%)**			
	Anxiety disorders	99 (99)	
	Trauma- and stressor-related disorders	90 (90)	
	Mood disorders	85 (85)	
	Disruptive, impulse control, and conduct disorders	44 (44)	
	Personality disorders	42 (42)	
	Somatic symptom and related disorders	33 (33)	
	Substance-related and addictive disorders	23 (23)	
**Primary treatment paradigm, n (%)**			
	Cognitive behavioral	69 (69)	
	Interpersonal	10 (10)	
	Existential or humanistic	7 (7)	
	Family systems	6 (6)	
	Psychodynamic or analytic	8 (8)	

^a^LMHC: licensed mental health counselor.

^b^LPC: licensed professional counselor.

^c^LCSW: licensed clinical social worker.

^d^LMFT: licensed marriage and family therapist.

#### Perceptions on Homework

Most therapists (94/100, 94%) reported that they generally asked their clients to practice therapeutic skills and exercises for homework *some* to *most of the time* (mean ranking 3.51, SD 0.61). Most therapists (73/100, 73%) felt that homework is *moderately* to *extremely* important to improving their clients’ outcomes (mean ranking 4.08, SD 0.93). Most therapists (69/100, 69%) also believed that it is *moderately* to *extremely* important for them to know the results of their clients’ homework assignments (eg, whether they completed them and how it went).

#### Barriers to Therapists Using Homework With Clients

Therapists perceived having difficulty getting clients to complete assignments (mean ranking 2.66, SD 0.79), not wanting to overwhelm or distress clients (mean ranking 2.05, SD 0.93), and being too busy or not having time (mean ranking 2.01, SD 0.90) as *minor* to *moderate* barriers to them using homework with their clients. Therapists perceived forgetting to assign homework (mean ranking 1.84, SD 0.90), not knowing what to assign (mean ranking 1.72, SD 0.71), not knowing how to address clients not completing assignments (mean ranking 1.69, SD 0.84), not knowing how to assign homework (mean ranking 1.34, SD 0.59), and not being trained to assign homework (mean ranking 1.29, SD 0.59) as *minor* barriers to nonbarriers to them using homework with their clients.

#### Barriers to Clients Completing Homework Assignments

Therapists perceived clients having a busy or chaotic home life as a *moderate* barrier to clients completing homework assignments (mean ranking 3.00, SD 0.80). Therapists also perceived clients forgetting about homework (mean ranking 2.86, SD 0.94), avoiding completing assignments due to distress or symptoms (mean ranking 2.66, SD 0.71), receiving little reward or reinforcement for completing assignments (mean ranking 2.10, SD 0.88), and viewing assignments as boring (mean ranking 2.06, SD 0.81) as *minor* to *moderate* barriers to clients completing homework. Furthermore, therapists perceived clients not knowing how (mean ranking 1.91, SD 0.77), what (mean ranking 1.88, SD 0.81), or why (mean ranking 1.73, SD 0.75) assignments should be completed as *minor* barriers to nonbarriers to their clients completing homework assignments.

#### New Adhere.ly Features to Prioritize for Therapists Seeing Adult Clients

With respect to adding new features to Adhere.ly, therapists primarily suggested adding self-report questionnaires or assessments (mean ranking 6.76, SD 1.46), integration with telemedicine platforms (mean ranking 5.18, SD 2.26), and therapy guides or treatment protocols (mean ranking 4.64, SD 2.31). These were followed by the ability to edit text language in SMS text message or email reminders (mean ranking 4.47, SD 2.08), customized exercises for their practice (mean ranking 3.97, SD 2.92), integration with EHR software (mean ranking 3.94, SD 2.38), incentives or rewards for clients (mean ranking 2.69, SD 1.82), integration with wearables (mean ranking 2.68, SD 1.89), and integration with other treatment software (mean ranking 1.67, SD 1.97).

#### New Adhere.ly Exercises to Prioritize for Therapists Seeing Adult Clients

Regarding adding new therapeutic exercises to Adhere.ly, therapists suggested prioritizing relaxation (eg, breathing, muscle relaxation, mindfulness, and grounding; mean ranking 7.38, SD 2.20) and self-monitoring (eg, journaling, emotions, thoughts, and behaviors; mean ranking 6.57, SD 2.08). These were followed by coping and emotion regulation (eg, cognitive reappraisal and acceptance; mean ranking 5.49, SD 2.33), behavioral activation (eg, scheduling pleasant and important activities; mean ranking 5.04, SD 2.18), interpersonal (eg, social skill training; mean ranking 4.88, SD 2.45), and cognitive (eg, restructuring, flexibility and reappraisal, and modifying core beliefs; mean ranking 4.64, SD 2.71) exercises. Lower-priority exercises included problem-solving (eg, simplification, visualization, and planful problem-solving; mean 3.37, SD 2.24), behavioral (eg, contingency management, stimulus control, and shaping; mean ranking 2.87, SD 2.19), exposure (eg, building imaginal and in vivo exposure hierarchies; mean ranking 2.59, SD 2.32), and couple (eg, communication, compromising, and problem-solving; mean ranking 2.17, SD 2.85) exercises.

## Discussion

### Principal Findings

This mixed methods study explored common barriers experienced by mental health therapists when implementing homework with their adult clients. This study also explored potential solutions to those barriers, including health technology such as Adhere.ly, a web-based platform designed to help therapists engage adult clients in homework. Therapists in this study provided their input on new features and exercises that would enable Adhere.ly to better meet their needs when working with this population.

Consistent with previous research, therapists reported facing substantial challenges when implementing homework with adult clients [6,7,9,14-21]. While most therapists rated homework as moderately to extremely important, only a small percentage reported consistent and successful implementation. These results align with previous findings [9-11]. The most used evidence-based therapy protocols, particularly cognitive behavioral interventions, rely on homework exercises, such as worksheets and coping skill practice [4,5], yet there are few solutions available to support therapists in carrying out this critical therapy component [14]. The significant barriers faced by mental health care providers and the lack of available tools to resolve these barriers have significant implications for the successful implementation of evidence-based therapies in real-world practice settings.

### Common Barriers to Homework

Primary barriers to therapists using homework with their adult clients differed significantly between the focus groups and survey in terms of barrier rankings.

While therapists and clients being too busy to assign or complete homework, respectively, was the top-ranked barrier in the focus groups, difficulty getting clients to complete assignments was the top-ranked therapist barrier in the surveys. Therapists being too busy to assign or review homework was the third priority-ranked barrier in the surveys. The emphasis on busyness and competing demands aligns with previous research [14,15]. Even when clients are motivated, therapists and clients juggle numerous responsibilities, commitments, and in-session priorities, which are frequently cited barriers to completing, assigning, and reviewing homework [15]. In the focus groups, therapist and client barriers were merged due to statements often referencing both therapist and client busyness, which is the mostly likely reason for the different rankings in the survey and focus groups. Furthermore, primarily clinic leaders identified therapist busyness as a barrier in the focus groups, and the surveys did not include clinic leaders.

Similarly, therapist perceptions that clients forget to complete their homework was ranked second in the focus groups and in the survey. Previous research [14,15] has identified clients forgetting as a primary barrier and emphasized the need for solutions that remind and encourage adult clients to complete homework given their other responsibilities. In contrast with child and adolescent clients, adults have no external reinforcement to stay focused on their therapy goals aside from their therapist, whom they may see at most once a week. Low rates of homework completion might be expected given that these barriers are coupled with clients finding assignments boring and receiving little reward or reinforcement for completing them, which was the third-ranked client barrier in the survey. Given that adult clients also struggle to remember what to do for homework and how to do it, solutions that remind clients to complete homework while providing easy-to-use and easy-to-access exercises with clear instructions have tremendous potential to overcome these common barriers to homework adherence.

The remaining barriers identified in the focus groups and the survey differed significantly. The third-ranked barrier in the focus groups was therapists struggling to find the right medium or tools with which to assign homework, followed by clients lacking the motivation to complete homework, clients receiving insufficient reinforcement for completing homework, clients avoiding homework due to distress, and other client barriers to homework (eg, lack of internet or smartphone or cognitive challenges). In contrast, the remaining survey priority-ranked categories were not wanting to overwhelm or distress clients and not knowing what to assign or how.

We attribute these differences to developing survey items using a combination of previous research [14,15] and a preliminary content analysis of the focus group results, using different samples for the focus groups and the survey, and the inclusion of clinic leaders in the focus groups who may have significantly different concerns from therapists (eg, emphasis on structural or administrative challenges). Furthermore, there is limited previous research in this area on which to base survey items, and prior work may not adequately capture barriers to implementing therapy homework. Indeed, the survey priority rankings were overall low for the client and therapist barrier question, indicating that the items selected for the survey were not representative of therapists’ concerns. These discrepancies emphasize the importance of this mixed methods study given the critical role of homework in evidence-based therapy protocols. The qualitative findings will be used to develop future survey items that are more reflective of therapists’ and clinic leaders’ experiences in real-world practice settings.

Focus group participants reported that therapists’ general therapeutic approach to addressing these challenges includes using motivational and reinforcement strategies to “meet clients where they are,” including completing homework in the session and adapting homework assignments so that they are less time-consuming to complete. Therapists reported using multiple web platforms, mobile apps, and other materials (eg, paper homework) to create, assign, and remind clients of homework. For example, therapists often use phone reminders, paper materials, and various mobile apps to follow homework assignments prescribed according to specific therapy protocols. However, this approach results in therapists having to use several different platforms and mediums to implement homework, which can be inefficient and time-consuming for both the therapist and client, leading to frustration and lower homework adherence. Participants reported high perceived value of an all-in-one solution to support them in this process. This perceived value highlights the importance of adapting Adhere.ly for use with adult clients.

### Adhere.ly as a Solution

#### Overview

Overall, participants viewed Adhere.ly as a potential solution to these challenges by offering a user-friendly, all-in-one solution. They felt that that Adhere.ly would likely reduce inefficiencies, increase homework completion rates, and be easy to use. Participants most frequently showed interest in Adhere.ly’s SMS text message or email reminder feature. This feature allows clients to simply click the link from their SMS text messages or email and complete brief therapy exercises.

Most participants stated that Adhere.ly would be helpful for a wide range of therapists and clients while also believing that its utility would depend on therapist and client demographics. The most frequently mentioned concern in this domain was that some populations might not have access to the internet or cell phones with data plans and some clients or therapists might need to be technologically savvy to use the platform. As a result, Adhere.ly may resolve barriers to homework for some but not all therapist and client populations. Some therapists suggested that generations who grew up around these technologies might find SMS text message–based reminders and exercises especially motivating and helpful. Therapists and clients who did not grow up around these technologies might find learning a new application burdensome or less engaging. Additional training and support resources, while alleviating some barriers, would not address underlying attitudes toward or existing patterns of technology use [34].

#### Suggested Features and Exercises

Although the survey and focus groups identified similar features and exercises to add to make Adhere.ly compatible with adult clients, there were significant differences in the extent to which these features and exercises were emphasized by both samples. Regarding features, the survey respondents ranked enhanced assessment, integration with telemedicine platforms (eg, Doxy.me), therapy guides, customizable reminders, and EHR integration as the top 5 most important features to add. Focus group participants emphasized a demonstration video to orient clients and therapists regarding the application, EHR integration, gamification, and custom reminders as the most important features to add. We suspect that these differences are largely due to the focus group sample including clinic leaders because all the focus group participants who emphasized the demonstration video and EHR integration and most of the participants who mentioned gamification were clinic leaders. In future studies exploring the utility of health technology to enhance the implementation of evidence-based therapies, we will include clinic leaders in both the surveys and focus groups and ensure that extensive demographic and professional information is collected in both samples.

Regarding requested therapy exercises, evidence-based therapy protocol homework assignments (eg, mindfulness-based therapies and trauma-focused interventions) were most frequently requested, followed by specific coping skill assignments (eg, journaling). Eye movement desensitization and reprocessing, cognitive processing therapy, and dialectical behavior therapy were the most frequently referenced interventions, reflecting a high need for trauma-focused homework solutions. The survey options were worded differently than the focus group exercise categories, but the results were fairly similar across the samples. Survey respondents also emphasized mindfulness and relaxation-based exercises, self-monitoring exercises, coping and emotion regulation, behavioral activation, interpersonal skills, and cognitive exercises as the top-ranked exercises to add. Given that 69% (69/100) of the survey respondents identified as cognitive behavioral therapists, it makes sense that the most requested exercises aligned with second- or third-wave cognitive behavioral interventions. Focus group participants were more likely to discuss the overall need for including evidence-based therapy protocol homework with an emphasis on mindfulness-based and trauma-focused interventions.

### Limitations

While this study offers valuable insights into common barriers faced by therapists to implementing homework with their adult clients and how health technology solutions such as Adhere.ly might help, there are some limitations to consider. This study included a small sample of therapists and clinic leaders for the focus groups and a separate sample of therapists for the survey. Recruitment was impacted by the COVID-19 pandemic, which led to smaller-than-recommended focus groups; however, this study was conducted during a period of unprecedented need for telehealth solutions, which we believe is a strength.

While efforts were made to ensure diversity in this sample, the findings may not reflect all mental health care providers, and true integration of the qualitative and quantitative findings would require at least some overlap in the survey and focus group samples. Furthermore, this study did not adequately assess therapist and clinic leader demographics to match the demographics collected in the survey, which will be corrected in future studies. Lack of inclusion of this information impacted our ability to assess how participants’ contexts may have influenced their responses. Specifically, the therapists and clinic leaders who consented to participate may have greater comfort with or interest in health technology solutions than the average mental health clinician.

In addition, these data were obtained using focus groups and self-report assessments, which are subject to inherent biases and limitations. While we aimed to reduce the potential for researcher bias, as described in the Methods section, all researcher contributions are also subject to inherent biases and limitations. This study aimed to assess therapist and clinic leader perceptions on Adhere.ly’s utility for adult-serving clinicians given that its utility for therapists serving children and families has been previously assessed. As a result, the findings cannot be generalized to therapists working across all populations and settings.

### Conclusions

This study identified primary challenges that therapists face while implementing homework in adult-serving mental health outpatient treatment. The findings indicate that, with the additional features and therapeutic exercises requested by respondents, Adhere.ly has strong potential to address some of these challenges and increase overall homework completion rates, which would theoretically support improved clinical outcomes.

The results of this study were used to inform the user-centered development of the requested features and exercises, which can be accessed freely through the Adhere.ly platform. However, the broader implications of this study are that it was conducted at a time of unprecedented need for telehealth solutions and, at present, evidence-based therapies are inadequately implemented in real-world practice settings due to the aforementioned challenges. There is incredibly limited research on evidence-based therapy homework implementation despite its clear implications for the implementation science literature.

Ongoing research is exploring the feasibility and effectiveness of using Adhere.ly in mental health therapy practice settings. Specifically, we are evaluating its ability to contribute to improved homework implementation and adherence and, in turn, clinical and functional outcomes. Future studies will also address the limitations of this study through continued refinement of Adhere.ly and study methodology. Overall, this study contributes to a growing but still small body of literature on therapy homework implementation and highlights the importance of innovative technology solutions that address common challenges experienced by therapists.

## Appendix

Multimedia Appendix 1 Adhere.ly feature categories.

Multimedia Appendix 2 Health care provider survey.

## Abbreviations

CBT: cognitive behavioral therapy
CFIR: Consolidated Framework for Implementation Research
COREQ: Consolidated Criteria for Reporting Qualitative Research
EHR: electronic health record
ITIM: Integrated Technology Implementation Model
