# Supplementary material for: Expanding a Health Technology Solution to Address Therapist Challenges in Implementing Homework With Adult Clients: Mixed Methods Study
Source: JMIR Hum Factors. 2024 Dec 12;11:e56567. doi: 10.2196/56567 (PMC11671782; doi:10.2196/56567)
Supplement: Multimedia Appendix 1 [file humanfactors_v11i1e56567_app1.docx]

## Multimedia Appendix 1

Table 3. Adhere.ly Features Categories

| Interview Question and Code Names | References | | |
| --- | --- | --- | --- |
|  | Total | Therapists | Leaders |
| **What challenges have you experienced while trying to engage clients in between-session exercises** | **44** | **16** | **28** |
| Clients and therapists are too busy to complete, assign, or review homework | 9 | 2 | 7 |
| Clients forget to complete homework | 8 | 3 | 5 |
| Clients and therapists struggle to find the right platform or medium for homework | 7 | 3 | 4 |
| Clients lack motivation or discipline to complete homework consistently | 7 | 4 | 3 |
| Clients receive insufficient instructions or rationale for completing homework | 6 | 3 | 3 |
| Clients avoid homework due to anticipated distress | 4 | 1 | 3 |
| Clients experience SES-related and or cognitive barriers to homework completion | 3 | 0 | 3 |
| **What strategies have you used to overcome these challenges** | **57** | **14** | **43** |
| Adapting treatment plan to meet clients where they are at | 14 | 7 | 7 |
| Clients set their own reminders either electronically or in written form | 3 | 0 | 3 |
| Accountability through email, text message, or phone | 2 | 1 | 1 |
| Applications and web platforms to send and track homework | 15 | 2 | 13 |
| Motivational and reinforcement strategies to encourage homework completion during session | 23 | 4 | 19 |
| **How well do you think Adhere.ly might address challenges to engaging patients in homework** | **143** | **64** | **79** |
| Adhere.ly is easy to use and would appeal to a wide range of therapists and clients | 27 | 12 | 15 |
| Adhere.ly might not resolve internal motivational barriers to homework completion | 4 | 4 | 0 |
| Adhere.ly would increase the efficiency of therapy | 23 | 11 | 12 |
| Adhere.ly can capture treatment data, such as symptom distress and homework adherence | 9 | 1 | 8 |
| Adhere.ly would easily fit into therapist's in-session workflow | 11 | 4 | 7 |
| Adhere.ly allows for custom exercises but also includes stock exercises | 2 | 1 | 1 |
| Adhere.ly's reminders feature would improve homework completion rates | 16 | 1 | 15 |
| Clients will be more engaged due to receiving reminders from their therapist as opposed to an independent app | 5 | 4 | 1 |
| Use of text-based communication will increase homework completion | 6 | 1 | 5 |
| The utility of Adhere.ly will vary by client demographics and clinical needs | 28 | 18 | 10 |
| The utility of Adhere.ly will vary by provider demographics and theoretical orientation | 12 | 7 | 10 |
| **4. What kinds of changes should we make to Adhere.ly so it will work in your practice** | **144** | **46** | **86** |
| **4a. Which therapeutic exercises should we add to support therapists and clients** | **81** | **47** | **34** |
| Homework from evidence-based protocols | 40 | 15 | 25 |
| Mindfulness and Stress Reduction | 11 | 6 | 5 |
| Trauma-focused EBP worksheets | 8 | 2 | 6 |
| DBT | 5 | 1 | 4 |
| Behavioral Activation | 4 | 3 | 1 |
| Safety Planning | 3 | 0 | 3 |
| CBT | 3 | 2 | 1 |
| ACT | 2 | 0 | 2 |
| PCIT exercises | 1 | 0 | 1 |
| exposures for phobias | 1 | 1 | 0 |
| Smoking cessation | 1 | 0 | 1 |
| Motivational Interviewing | 1 | 0 | 1 |
| Homework for specific skills or strategies | 17 | 10 | 7 |
| Journaling | 7 | 7 | 0 |
| Creative coping (i.e., drawing, painting) | 3 | 0 | 3 |
| Recovery plan | 2 | 0 | 2 |
| Positive Psychology or Strengths-Based Coping (i.e., strengths, affirmations) | 2 | 0 | 2 |
| Movement breaks for anxiety | 1 | 1 | 0 |
| psychoeducation | 1 | 1 | 0 |
| Emotion regulation | 1 | 1 | 0 |
| Homework for specific client demographics (i.e., children, couples, families) | 6 | 6 | 0 |
| Emotion regulation games for kids | 3 | 3 | 0 |
| Couples | 2 | 2 | 0 |
| Communication styles for families, couples, children | 1 | 1 | 0 |
| Behavior, habit, or activity tracking | 5 | 3 | 2 |
| Health behavior tracking | 2 | 2 | 0 |
| Medication adherence | 1 | 0 | 1 |
| Recovery group attendance (e.g., NA AA) | 1 | 0 | 1 |
| Behavior trackers with visuals | 1 | 0 | 0 |
| Homework for specific mental health conditions | 5 | 5 | 0 |
| Sexual dysfunction | 1 | 1 | 0 |
| Depression | 1 | 1 | 0 |
| ASD | 1 | 1 | 0 |
| ADHD | 1 | 1 | 0 |
| Anxiety | 1 | 1 | 0 |
| Personalized or custom exercises | 5 | 5 | 0 |
| Clickable personalized coping plans | 4 | 4 | 0 |
| Custom exercises | 1 | 1 | 0 |
| Single item rating scales | 3 | 3 | 0 |
| **4b. What features should we add to support therapists and adult clients** | **63** | **11** | **52** |
| Add a demo video or other interactive walk-through for clients and therapists | 14 | 2 | 12 |
| Integrate with other platforms e.g., EHR, telehealth | 12 | 0 | 12 |
| Enhanced audiovisual features (i.e., games, gamification of homework, more color and visuals) | 11 | 3 | 8 |
| Expand the reminders features (i.e., reoccurring notifications, customization, parent reminders) | 10 | 5 | 5 |
| Add re-occurring reminders with one click | 3 | 1 | 2 |
| Parent notifications for their childrens' exercises | 2 | 2 | 0 |
| Appointment reminders | 2 | 2 | 0 |
| Reminders for incomplete homework | 1 | 0 | 1 |
| Notification to the therapist when client completes assignment | 1 | 1 | 0 |
| Automated outcome tracking (i.e., set reoccurring assessments) | 1 | 1 | 0 |
| Expand registration options with more customization and client self-registration | 8 | 0 | 8 |
| Client self-registration | 3 | 0 | 3 |
| Customized communication preferences for homework reminders | 3 | 0 | 3 |
| Add more client identifiers so accounts don't get confused | 1 | 0 | 1 |
| Feature to add family, couple, and group clients | 1 | 0 | 1 |
| Add an e-Consent | 5 | 0 | 5 |
| Features mentioned that did not fit in other categories | 3 | 1 | 2 |
| Client feedback on exercises | 1 | 1 | 0 |
| Export client results to PDF | 1 | 0 | 1 |
| 6th and 7th grade reading level for all content | 1 | 0 | 1 |
