# Supplementary material for: Expanding a Health Technology Solution to Address Therapist Challenges in Implementing Homework With Adult Clients: Mixed Methods Study
Source: JMIR Hum Factors. 2024 Dec 12;11:e56567. doi: 10.2196/56567 (PMC11671782; doi:10.2196/56567)
Supplement: Multimedia Appendix 2 [file humanfactors_v11i1e56567_app2.docx]

## Multimedia Appendix 2

### Provider Survey

#### Informed Consent

[Insert IRB approved consent form]

#### Screening

1. What is your age, in years? ________

1. What kind of mental health therapist are you?

1 = Mental Health Counselor (e.g., LMHC, LPC)

2 = Social Worker (e.g., LCSW)

3 = Marriage and Family Therapist (e.g., LMFT)

4 = Psychologist (e.g., PhD, PsyD)

5 = Behavior Analyst (e.g., ABA)

6 = Other mental health therapist: _________

0 = I am not a mental health therapist

1. What is the highest degree you’ve obtained?

1 = Masters degree (e.g., MSW)

2 = Doctoral degree (e.g., PhD, PsyD)

0 = I don’t have a masters or doctoral degree

1. Which state do you primarily practice in?

Select from the drop-down menu.

#### Personal Demographics

1. Do you identify as male or female?

0 = Male

1 = Female

1. How do you identify racially?

1 = White

2 = American Indian/Alaska Native

3 = Asian

4 = Native Hawaiian or Pacific Islander

5 = Black or African American

6 = Multiracial

1. Are you of Hispanic, Latinx, or Spanish origin?

0 = No

1 = Yes

#### Professional Demographics

1. What age group(s) do you primarily treat? (Select all that apply)

- Children (0-10 yrs old)
- Adolescents (11-17 yrs old)
- Adults (18-64 yrs old)
- Older adults (65+ yrs old)

1. What mental health disorders do you commonly treat? (Select all that apply)

- Anxiety disorders
- Mood disorders
- Trauma- and stressor-related disorders
- Substance-related and addictive disorders
- Personality disorders
- Disruptive, impulse control, and conduct disorders
- Somatic symptom and related disorders
- Other (Please specify): ________________

1. How would you describe your clinic or organization?

1 = Individual practice

2 = Network of providers or small clinic

3 = Hospital or large clinic

4 = School (K-12)

5 = College or university

6 = Government agency (e.g., Veterans Affairs)

1. Is your clinic or organization for-profit or non-profit?

1 = Non-profit

2 = For-profit

1. How are you primarily reimbursed for your telemedicine services?

1 = Public Insurance (Medicare, Medicaid)

2 = Private Insurance

3 = Client out-of-pocket

1. Which treatment paradigm do you primarily follow?

1 = Cognitive-Behavioral

2 = Existential/Humanistic

3 = Family Systems

4 = Interpersonal

5 = Psychodynamic/analytic

6 = Social Learning

#### Homework

1. In general, how often do you ask your clients to practice therapeutic skills and exercises between therapy sessions (i.e., for homework)?

1 = Never

2 = Seldom

3 = Some of the time

4 = Most of the time

1. How important do you think homework is to improving your clients’ outcomes?

1 = Not at all

2 = Slightly

3 = Somewhat

4 = Moderately

5 = Extremely

1. How important is it for you to know the results of your clients’ homework assignments (e.g., whether they completed it, how it went, )?

1 = Not at all

2 = Slightly

3 = Somewhat

4 = Moderately

5 = Extremely

1. How much of a barrier is each of the following to you using homework with your clients?

(1 = Not a barrier; 2 = Minor barrier; 3 = Moderate barrier; 4 = Significant barrier)

Having difficulty getting clients to complete assignments

Not knowing what to assign

Not knowing how to assign homework

Forgetting

Being too busy/not having time

Not knowing how to address clients not completing assignments

Not wanting to overwhelm or distress clients

Not being trained to assign homework

1. How much of a barrier is each of the following to your clients completing homework assignments?

(1 = Not a barrier; 2 = Minor barrier; 3 = Moderate barrier; 4 = Significant barrier)

Avoiding completing assignments due to distress or symptoms

Not knowing what to do

Not knowing how to do it

Not knowing why they should do it

Forgetting

Having a busy/chaotic home life

Viewing assignments as boring

Receiving little reward/reinforcement for completing assignment

#### New Adhere.ly Exercises and Features

1. Please rank the following types of therapeutic exercises in the order that we should prioritize adding them to Adhere.ly to meet **your** needs when seeing your adult clients*.*

Relaxation (e.g., breathing, muscle relaxation, mindfulness, grounding)

Interpersonal (e.g., social skills training)

Self-monitoring (e.g., journaling, emotions, thoughts, behaviors)

Behavioral activation (e.g., scheduling pleasant and important activities)

Exposure (e.g., building hierarchies, imaginal, in-vivo)

Behavioral (e.g., contingency management, stimulus control, shaping)

Problem solving (e.g., simplification, visualization, SSTA, planful problem solving)

Coping and emotion regulation (e.g., cognitive reappraisal, acceptance)

Cognitive (e.g., restructuring, flexibility & reappraisal, modifying core beliefs)

Couples (e.g., communication, compromising, problem solving)

1. Please feel free to suggest additional therapeutic exercises we might have missed (optional)

[Paragraph Text Box]

1. Please rank the following features in the order that we should prioritize adding them to Adhere.ly to meet **your** needs when seeing your adult clients*.*

Self-report questionnaires/assessments

Integration with telemedicine platforms

Integration with Electronic Health Record (EHR) software

Therapy guides/treatment protocols

Ability to edit text language in text/email reminders before sending

Integration with wearables (e.g., smart watches) to detect distress and suggest intervention

Incentives/rewards for clients (e.g., games, badges)

Integration with other treatment software (e.g., EMDR)

Customized exercises and communications for my practice

1. Please feel free to suggest additional features that we might have missed (optional)

[Paragraph Text Box]
